# Effect of prolonged cooking on pro-vitamin A levels of biofortified East African highland bananas

**DOI:** 10.1080/21645698.2025.2529637

**Published:** 2025-07-09

**Authors:** Stephen Buah, Janefer Kiwummulo, Jean-Yves Paul, Joel Walugembe, Jackline Wegesa, Robooni Tumuhimbise, Wilberforce Tushemereirwe

**Affiliations:** a National Banana Research Program, National Agricultural Research Laboratories, Kawanda, National Agricultural Research Organisation, Entebbe, Uganda; b Department of Biological Sciences, Faculty of Science, Kyambogo University, Kampala, Uganda; c Centre for Agriculture and the Bioeconomy, Queensland University of Technology, Brisbane, QLD, Australia

**Keywords:** Biofortification, micronutrient, nutritional deficiency, vitamin A, α-carotene, β-carotene

## Abstract

Genetically modified East African highland bananas (EAHBs) with elevated levels of pro-vitamin A (pVA) have been developed to address vitamin A deficiency (VAD) in Uganda. The green, mature fruits of EAHBs are traditionally prepared into “matooke,” a soft, savory dish made by peeling, steaming, and mashing the fruit. Biofortifying such a culturally embedded food offers an effective approach to improving nutrition, particularly among vulnerable populations. Field trials of pVA-biofortified EAHBs have been conducted at four locations across Uganda, with data collection nearing completion. However, the effect of prolonged cooking, a common traditional preparation method, on pVA carotenoid retention had not been evaluated. This study investigated the retention of β-carotene equivalents (β-CE) during extended cooking in two elite events, each of hybrid M9 and Nakitembe. After 1 h of cooking, β-CE concentrations declined significantly in both wild-type and biofortified lines. Further cooking, up to 8 h, did not result in additional significant losses. By the eighth hour, β-CE levels remained above the target threshold of 20 µg/g dry weight (DW) in one M9 event (21.7 µg/g DW) and both Nakitembe events (28.7 and 27.6 µg/g DW), compared to 12.8 and 17.6 µg/g DW in their respective wild-type controls. This confirms that the biofortified bananas can deliver meaningful nutritional benefits under customary preparation methods. These results are not only pivotal for selecting final lead and backup events but also provide compelling evidence of trait stability, further supporting the case for deployment of biofortified EAHBs to improve public health outcomes in East Africa.

## Introduction

Vitamin A (VA) is an essential fat-soluble micronutrient required for normal vision, strong immune system, reproduction, and the maintenance of healthy skin and epithelial tissues.^1,2^ As it cannot be synthesized endogenously, VA must be obtained through the diet, either as preformed VA (retinol and retinyl esters) from animal-derived foods or as pro-vitamin A carotenoids (pVAC) from fruits and vegetables. Insufficient intake of VA or its precursors leads to vitamin A deficiency (VAD), which is associated with a range of adverse health outcomes, including blindness, impaired cognitive development, and compromised immune function.^3^

The problem of VAD is prevalent in low- and middle-income countries partly due to over-dependence on a few starchy staple crops. Despite ongoing interventions, it is estimated that more than 2 billion people worldwide are affected VAD globally.^4^ In low-income settings, nearly 30% of children under the age of five are deficient in vitamin A.^5^ Biofortification of staple crops offers a sustainable and long-term strategy to combat micronutrient deficiencies in affected communities. This can be achieved through agronomic practices, conventional breeding, genetic modification, and more recently gene editing.^6–9^

Against this background, the National Agricultural Research Organisation, in collaboration with partners at Queensland University of Technology, developed biofortified East African highland banana (EAHB) varieties hybrid M9 (M9) and Nakitembe, with enhanced fruit pro-vitamin A content. This was achieved through genetic modification using a single *MtPsy2a* transgene, derived from the Fe’i banana “Asupina,”^10,11^ which effectively increased fruit pVA concentrations to exceed a target threshold of 20 µg/g DW β-carotene equivalents (β-CE).^9^ This biofortification target was determined based on several key factors: (a) a conservative bioconversion ratio of 6:1 for α- and β-carotene to retinol in humans^12^; (b) the goal of meeting at least 50% of the Estimated Average Requirement (EAR) for VA^13^; (c) an assumed daily consumption of 500 g of cooked EAHB, locally known as “matooke”^14^; and (d) adjustments for dry matter and processing losses, accounting for 75% moisture content and 25% losses during steaming and boiling.^15^ In comparison, β-CE concentration in non-biofortified cooking banana fruit typically range from 4.5 to 10.2 µg/g DW, depending on the cultivar.^15^ The established target is approximately four times higher than that found in commonly consumed varieties such as M9.

More than 100 million people across tropical and subtropical regions of Africa rely on banana and plantain as a staple food and a key component of food security.^16^ In Uganda, EAHB make up a significant portion of the diet for both rural and urban populations.^17^ These bananas are typically consumed as a local dish known as “matooke,” made by peeling mature green fruits, wrapping them in banana leaves, and placing them over a small volume of water in large aluminum saucepans. They are then steamed over an open wood fire for 30 min to 1 h. The cooked fruits are usually mashed into a thick, smooth paste called “matooke,” which is eaten with various accompaniments such as groundnut sauce, vegetables, meat or fish.^18^ “matooke” is traditionally prepared and served both in households and at large family, community, and social gatherings such as weddings and funerals. The widespread popularity of EAHB therefore presents a strategic biofortification pathway to reach populations most affected by micronutrient deficiencies.

Following the successful field evaluation and selection of elite biofortified EAHB lines,^10^ multi-location regulatory field trials were conducted to generate comprehensive data on agronomic performance, biochemical characteristics, soil composition, and fruit proximate parameters. These trials are now nearing completion. However, although cooking time is known to affect pVAC retention, data on the impact of prolonged cooking beyond 1 h are lacking, despite the common practice of keeping “matooke” on the fire for much of the day. Previous studies have reported pVAC losses of 25–60 percent during boiling or steaming, based on cooking durations ranging from 30 min to 1.5 h, respectively.^15,19,20^ In many Ugandan households, “matooke” is prepared once and kept hot over a wood fire throughout the day to serve both lunch and dinner, typically to maintain its softness.^18,21^ However, the effect of such extended cooking on pVAC content has not been previously evaluated.

Understanding the stability of pVACs under prolonged cooking is essential for developing clear dietary recommendations and maximizing the nutritional impact of pVA-biofortified bananas in efforts to combat VAD. This study therefore investigated the retention of β-CE in biofortified banana cooked for varying durations over an 8-h period. The findings provide critical evidence to inform the effective use of biofortified “matooke” in addressing micronutrient deficiencies in Uganda.

## Materials and Methods

### Sample Collection and Preparation

The source of fruits used in this study were from a multi-location confined field trial (ML-CFT) of elite events of EAHBs, which were established at two of the four locations in Uganda. Each of the two cultivars had five independent events and two wild-type controls planted in a randomized block design, each line represented by 20 copies/clones. Two transgenic events, each of M9 (M9–12083 and M9–12141) and Nakitembe (NKT-12468 and NKT-12431), and their respective non-transgenic (wild-type) control lines, were selected based on the availability of bunches for harvest at the same time. Two plants (biological replicates) that flowered at the same time for each of the selected events were tagged with ribbons for sample collection at physiological maturity.

Harvested bunches were packed and transported from the fields to the National Agricultural Research Laboratories, Kawanda in accordance with the National Biosafety Committee (NBC) terms and conditions (Decision No. NBC/2/2018). For the uncooked sample, one fruit from each of the top, middle and bottom parts of the bunch was peeled and the pulp cut into 1-cm^3^ slices. Equal amounts (at least 10 g) of each portion were combined into a composite sample and placed in labeled petri dishes followed by freezing at −80°C until further processing. For the cooked samples, at least 12 of fruits were collected from the top, middle, and bottom clusters of the same bunch, peeled and wrapped in green leaves sourced from the non-transgenic control M9 and Nakitembe plants. These were divided into two technical replicates, each placed in a labeled stainless-steel saucepan and steamed over an open wood fire for 1 h. After this initial cooking, the pulp was mashed into a paste, and samples were collected and placed in labeled petri dishes. Cooking then resumed, with additional samples collected at 2, 4, 6, and 8 h. After each sampling, the remaining “matooke” was rewrapped and returned to the fire. All cooked samples were cooled to room temperature and frozen at −80°C. Both raw and cooked frozen samples were then freeze-dried at −50°C under a vacuum of 0.1 m Bar for 72 h in an Alpha 1–4 LSC Basic freeze dryer (Martin Christ, Osterode am Harz, Germany). Freeze-dried samples were ground to a fine powder using ceramic mortars and pestles. The powdered samples were then stored in the −80°C freezer until required for analysis.

### Carotenoid Extraction

Total carotenoids were extracted as previously described by Buah et al.^22^ Briefly, 200 mg of freeze-dried powder for each sample was weighed in a 2 mL screw-capped tube containing a 5 mm diameter stainless steel bead. The powder was further homogenized using a Mini Bead beater (Biospect products) at 30 cycles per second for 30 s. Acetone (1 mL) was added to each tube containing the sample, mixed by bead-beating and transferred into each respective labeled 15 mL Falcon tube containing 100 µL of a 1 mg/mL α-tocopherol acetate internal standard solution. The mixture was vortexed for 30 s (sec), centrifuged at 3880 x*g* for 5 min, and the supernatant collected into a new 15 mL tube. Extraction with acetone was repeated three times. The organic phase was then separated from the inorganic phase using a solution of petroleum ether:diethyl ether (2:1; v/v) and 1% sodium chloride solution. The organic phase was collected into two new 1.5 mL tubes for each sample, vacuum-dried for 2–3 h, and stored at −20°C prior to high-performance liquid chromatography (HPLC) analysis.

### HPLC Analysis

The vacuum dried carotenoid extracts were re-suspended in 100 µL of solvent C (Methanol:*tert*-Butyl methyl ether, 1:1 v/v), vortexed and pulse spun. Resuspended extracts from two tubes of the same sample were combined into a single tube, briefly vortexed and centrifuged at 10,000 x*g* for 5 min at room temperature before a 100 µL aliquot of the resulting supernatant was transferred into a 250 µL glass insert placed inside an amber HPLC vial for analysis.

Carotenoids were quantified using an Agilent 1100 series HPLC system equipped with a diode array detector set at 450 nm for pVAC and 285 nm for the α-tocopherol acetate internal standard and essentially as described by Buah et al.^22^ Briefly, 10 μL of each extract was injected and separated on a C30 reverse-phase column (4.6 × 250 mm, 3 μm particle size) coupled to a C30 guard cartridge (4.0 × 23 mm, 5 µm; YMC, Kyoto, Japan). The mobile phases consisted of solvent A (Methanol:*tert*-Butyl methyl ether, 1:1 v/v) and solvent B (Methanol:*tert*-Butyl methyl ether:distilled water, 5:1:1 v/v/v). β-carotene equivalents (β-CE) was calculated from the total pVAC content and expressed in µg/g on a dry weight basis.

### Data Analysis

Data were analyzed for significant differences in β-CE means across cooking time points using one-way ANOVA. Significant ANOVA was followed by Mann-Whitney’s multiple comparison test for β-CE proportions, and by Tukey’s multiple comparison test for β-CE concentrations, as implemented in the PAST software version 4.02. A *p*-value of 0.05 was interpreted as statistically significant. Graphs illustrating trends in β-CE levels during prolonged cooking were generated using GraphPad Prism version 9.3.1.

## Results

### Profile and composition of pro-vitamin A carotenoinds in raw and cooked banana fruits

**Figure 1.**
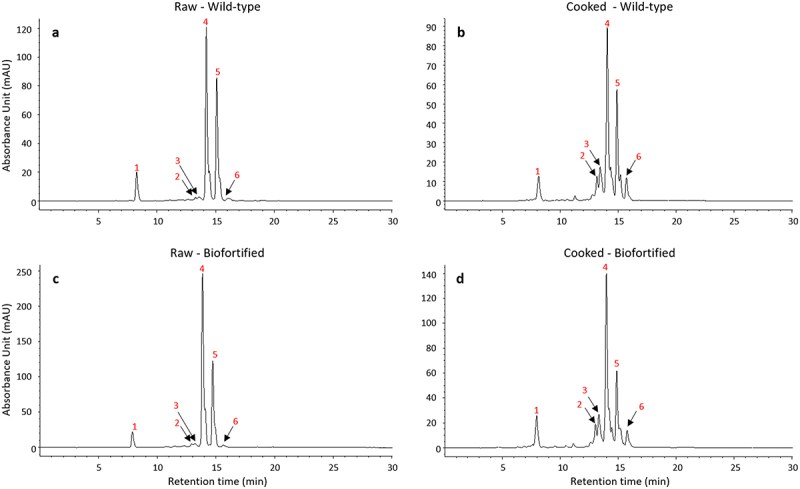
HPLC chromatographs of raw and cooked Nakitembe wild-type and biofortified (NKT-12468) banana fruit. (a) raw and (b) cooked wild-type; (c), raw and (d), cooked biofortified. Peaks 1, 4 and 5 are lutein, α-carotene and all trans-β-carotene, respectively. Peaks 2, 3 and 6 are isomers of α- and β-carotene more abundant following cooking. Cooking was sustained for 8h.

The general carotenoid profile of wild-type and biofortified Nakitembe fruit was initially examined to assess differences between raw and cooked samples. HPLC chromatograms of raw and cooked fruit from both wild type and the biofortified transgenic line NKT-12468 showed similar retention times and peak shapes, indicating comparable carotenoid composition. However, a consistent reduction in total carotenoid content was observed following cooking (Figure 1). Three major carotenoids were identified across all samples: lutein (peak 1), α-carotene (peak 4), and all-trans β-carotene (peak 5). In addition, peaks 2, 3, and 6 corresponded to isomers of α- and β-carotene, which became more prominent after cooking (Figure 1b,d).

**Figure 2.**
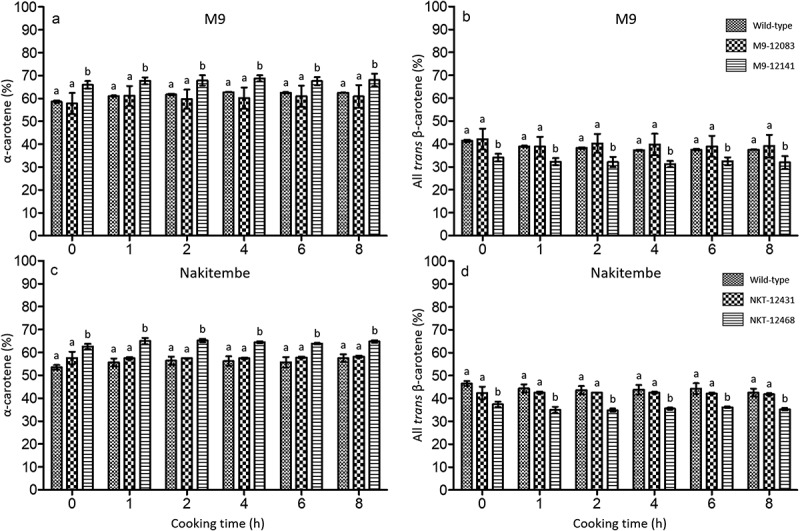
Variation in the composition of pVACs α- and all-*trans-*β-carotene in wild-type versus biofortified bananas during cooking. a and b, proportion of α- and all *trans-*β-carotene in M9 wild-type and biofortified lines M9 –12,083 and M9 –12,141; c and d, proportion of α- and all *trans-*β-carotene in Nakitembe wild-type and biofortified lines NKT-12431 and NKT-12468. Error bars represent mean ± SE of 2 biological replicates (clones) and 2 technical replicates of each. Different letters indicate statistically significant differences between groups based on Mann-Whitney pairwise comparisons (*p* < .05).

Following an 8-h traditional cooking regimen, the proportions of the main pVACs, α- and *trans-*β-carotene, in banana fruit were scrutinized. There were no significant differences (*p* > .05) in the proportion of α-carotene in the wild-type when compared to event M9–12083. In wild-type M9, the proportion of α-carotene increased from 58.6% in the raw sample (time 0) to an average of 62.5% over the entire cooking period (Figure 2a). In M9–12083, the α-carotene composition increased from 57.9% in the raw sample to 60.9% after 8 h of cooking. Event M9–12141, however, showed a significant difference in the proportion of α-carotene from that of the wild-type and event M9–12083, increasing from 66.0% in the raw sample to 68.1% after 8 h of cooking. Similarly, the proportion of all-*trans-*β-carotene did not differ significantly between the wild-type and event M9–12083. In the wild-type all-*trans*-β-carotene decreased from 41.4% in the raw sample to 37.5% after 8 h of cooking, while in M9–12083 all-*trans*-β-carotene decreased from 42,1% to 39.1% (Figure 2b). In contrast, raw fruit from event M9–12141 showed a significantly lower proportion of β-carotene compared to both the wild-type and event M9–12083 (Figure 2b), a difference that persisted throughout all stages of cooking. The all-*trans*-β-carotene content in M9–12141 also decreased from 34.0% in the raw sample to 31.9% by the end of the experiment (Figure 2b).

In Nakitembe, there was no significant difference in the proportions of α- and β-carotene between the wild type and event NKT-12431 at any stages of the cooking period (*p* > .05). Event NKT-12468, however, had significantly more α-carotene and corresponding less proportions of all-*trans-*β-carotene (*p* < .05) (Figure 2c and d). The proportion of α-carotene in the wild-type increased from 53.5% in the raw sample to an average of 57.5% over the entire cooking period, while all-*trans-*β-carotene reduced from 46.6% to 42.6% during the same period. In event NKT-12468, α-carotene increased from 62.6% to 64.7% during that time, while all-*trans-*β-carotene decreased from 37.5% to 35.3% between the raw and cooked samples, respectively (Figure 2c and d).

### Trend in β-carotene equivalents (β-CE) during cooking

**Figure 3.**
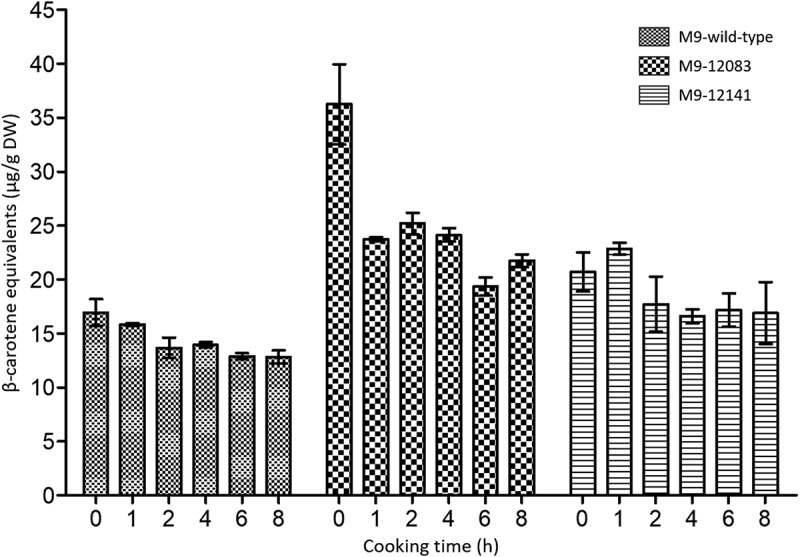
Effect of cooking time on β-CE concentration in ‘matooke’ made from M9 wild-type and biofortified events M9 –12083 and M9 –12141. β-CE levels were determined in the uncooked sample (time 0), followed by 1 h of cooking, and thereafter every 2 h up to the 8th hour. Error bars represent mean **±** SE of 2 biological replicates and 2 technical replicates of each. DW, dry weight.

The M9 lines showed a general decline in β-CE levels over the 8-h cooking period (Table 1). The β-CE levels in M9 wild-type decreased significantly (*p* = .03) from 16.9 µg/g DW in the uncooked sample to 12.8 µg/g DW in the cooked sample at 8 h, representing a 24.2% reduction over that time (Figure 3). In all samples, wild-type and biofortified, fluctuations in β-CE levels were observed across all cooking stages, probably due to sample inhomogeneity, although the overall trend was downward (Figure 3). Significant (*p* < .01) reductions in β-CE levels were observed in both biofortified M9 events. In event M9–12083, β-CE dropped sharply from 36.3 to 23.7 µg/g DW (35% loss) after 1 h of cooking. The levels then remained generally stable up to the 8th hour of cooking at which time the β-CE concentration retained in the sample was still above the target at 21.8 µg/g DW. In contrast, event M9–12141 showed no significant difference in β-CE content between the raw sample and those cooked for one or 2 h. Subsequently, significant (*p* < .05) reductions were detected in samples cooked for four to 8 h. Additional cooking between 4 and 8 h did not appear to further reduce carotenoids content in these samples (Figure 3). After 8 h of cooking the β-CE concentration in “matooke” made from event M9–12141 was only 16.9 µg/g DW but statistically significant compared to its wild-type counterpart that had 12.8 µg/g DW (*p* < .05).

**Table 1.** β-CE concentrations of two biofortified EAHB events and their wild-types during prolonged cooking.

Cultivar	Line ID	n	β-CE range (µg/g DW)	β-CE at 0h (µg/g DW)	β-CE at 8h (µg/g DW)	% reduction at 8h
M9	Wild-type	4	14.3–19.4	16.9	12.8	24.3
	12083	4	28.9–43.2	36.3	21.7	40.2
	12141	4	17.5–24.6	20.7	16.9	18.4
Nakitembe	Wild-type	4	14.2–27.6	20.9	17.6	15.8
	12431	4	40.2–68.5	53.8	28.6	46.8
	12468	4	46.1–64.8	55.3	27.6	50.1

n, biological replicate; β-CE, β-carotene equivalents; DW, dry weight.

In raw wild-type Nakitembe fruit, the β-CE concentration already averaged at 20.9 µg/g DW. As opposed to our observation with M9, cooking up to 8 h did not affect this value significantly. A slight reduction was recorded from the second hour of cooking and a further drop after 4 h to about 17 µg/g DW. This approximate 18.5% reduction in original β-CE concentration remained unchanged thereafter and up to the end of the experiment.

**Figure 4.**
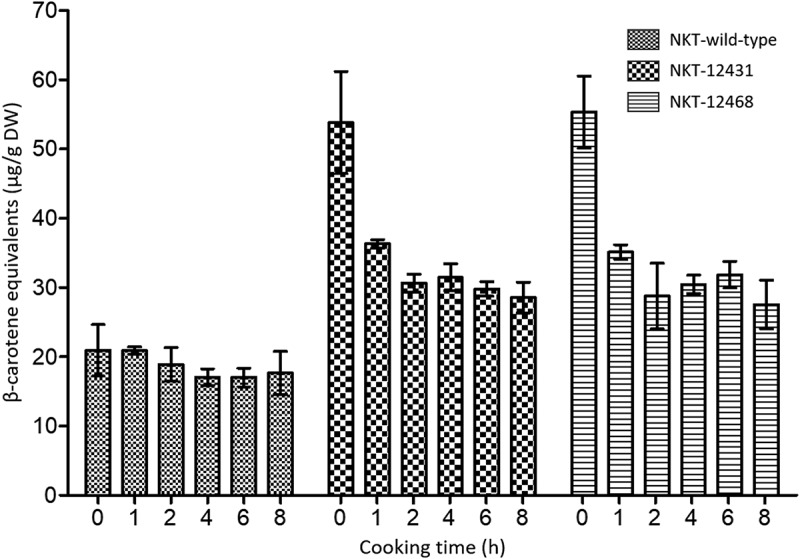
The effect of cooking time on β-CE concentration in ‘matooke’ made from Nakitembe wild-type and biofortified events NKT-12431 and NKT-12468. β-CE levels were determined in the uncooked sample (time 0), followed by 1 h of cooking, and thereafter every 2 h up to the 8^th^ hour. Error bars represent mean **±** SE of 2 biological replicates and 2 technical replicates of each. DW, dry weight.

Both uncooked biofortified Nakitembe lines had the highest β-CE levels with averages of 53.8 and 55.4 µg/g DW for events NKT-12431 and NKT-12468, respectively. A significant decrease (*p* < .05) in β-CE was observed during the first hour of cooking in both events with a reduction of 35.1% in event NKT-12431 and 36.6% in event NKT-12468 (Figure 4). The levels of β-CE continued to drop moderately at each time point, reaching nearly 50% losses by the eighth hour of cooking in both events. Prolonged cooking from 2 to 8 h did not have any significant effect on β-CE levels, which remained well above the target of 20 µg/g DW during the entire 8 h of cooking (Figure 4).

**Figure 5.**
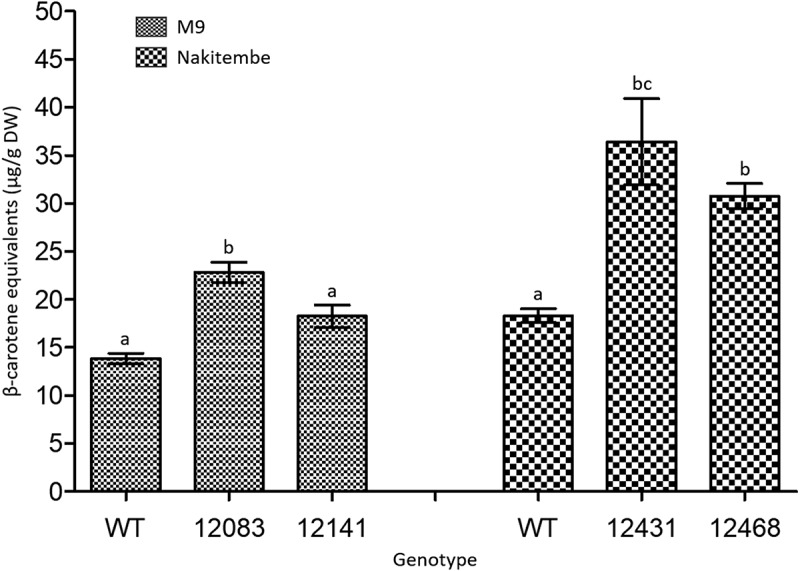
Average β-CE concentration across all cooking stage in wild-type and biofortified M9 and Nakitembe lines. Data represent mean β-CE levels across all cooking stages, from 1 to 8 h. Error bars represent mean **±** SE of 2 biological replicates and 2 technical replicates at each cooking stage (*n*=16). Different letters indicate statistically significant differences between groups based on Tukey’s multiple pairwise comparisons (*p* < .05). DW, dry weight.

To capture the practical nutritional value of each genotype under typical household preparation, β-CE concentrations were averaged across all cooking durations (1, 2, 4, 6, and 8 h). This integrated measure reflects the average pVA intake likely to be delivered, irrespective of cooking time. In the M9 background, at 22.8 µg/g DW only line M9–12083 retained significantly higher average β-CE levels than the M9 wild-type (*p* < .05) while exceeding the biofortification target (Figure 5). However, line M9–12141, with an average of 18.3 µg/g DW, did not differ significantly from the M9 wild-type (*p* = .65). In contrast, both Nakitembe events, NKT-12431 and NKT-12486, with 36.4 and 30.8 µg/g DW β-CE, exceeded the target by more than 82% and 54%, respectively (Figure 5). Both lines were significantly different from the wild-type (*p* < .01) but not from each other (*p=*0.39).

## Discussion

The current study investigated the effect of prolonged cooking on the retention of pVAC in genetically modified biofortified EAHBs. In this context, prolonged cooking refers to durations exceeding 1 h, a common practice in predominantly banana-eating communities in Uganda where “matooke” is cooked for at least 1 h, and after mashing, the food is maintained hot on simmering heat until serving.^23^ In addition, for traditional functions such as marriages and funerals, “matooke” is often kept on the fire for several hours or even overnight before serving.^18^ This cultural practice has prompted biosafety regulators and members of the public to question whether biofortified EAHBs would still meet the estimated average requirement (EAR) for vitamin A following such extended exposure to heat, which can degrade thermolabile compounds. To the best of our knowledge, this is the first report to examine β-CE retention in a biofortified crop over a prolonged cooking period of up to 8 h.

This study demonstrated that the most substantial losses of β-CE in both wild-type and biofortified bananas occurred within the first hour of cooking. This observation aligns with findings by Walugembe *et al*.,^20^ who reported β-CE losses of 53.9% in transgenic M9 and 28.6% in Nakitembe after 1.5 h of steaming. Beyond this point, observed fluctuations were not statistically significant enough and are more likely attributable to sample inhomogeneity rather than continued heat degradation. The initial sharp decline may be partially explained by the rapid isomerization of trans-β-carotene into its less bioactive forms, primarily 13-cis-β-Carotene, followed by 9-cis-β-carotene and other unidentified isomers in Fig. 1.^24,25^ Since cis-isomers contribute minimally to β-CE calculations, their formation results in an overall reduction in β-CE content after cooking, as also reported by Walugembe *et al*.^20^ Additionally, as the cooking method involved steaming the pulp above boiling water, it is possible that some carotenoids were lost through leaching into the water, as suggested by Mbabazi *et al*.^15^

This study also showed a gradual but not significant increase in the proportion of α-carotene with cooking in both wild-type and transgenic biofortified events. In contrast, β-carotene levels gradually declined over the same cooking period, with the reduction being more pronounced in M9. The greater decline in β-carotene is likely due to its higher sensitivity to heat, as β-carotene is more heat labile than α-carotene. It is important to note that β-carotene contributes more significantly to β-CE than α-carotene, and thus, reductions in its levels have a greater impact on total β-CE content, as observed in this study. The observed increase in α-carotene is supported by the findings of Bao *et al*.,^26^ who reported a similar trend during the cooking of carrots. Likewise, varying losses of β-carotene due to cooking have been reported in different types of vegetables^27^ and food matrices.^28^ These losses occur through oxidation and sterioisomerisation of β-carotene during thermal processing, ultimately leading to decreases in β-CE. The extent of these losses is influenced by both the cooking method and the nature of the plant matrix.

The observed fluctuations in β-CE concentrations may be attributed to sampling inconsistencies, particularly the difficulty in obtaining a homogenized tissue mixture representing equal proportions of fruit from the top, middle and bottom sections of the bunch at each of time points. Achieving uniform sampling was especially challenging given the physical nature of the cooked material, which consisted of hot, steamed fruits enclosed in banana leaves. As β-CE content may vary spatially within a bunch, failure to adequately homogenize the sample is likely to have introduced variability in the measured concentrations. Future studies could investigate whether sampling from a consistent positional section of the bunch, rather than compositing tissues from multiple locations, would reduce β-CE variability across time points during cooking.

The wild-type controls analyzed in this study exhibited higher β-CE concentrations than those previously reported. Specifically, raw M9 and Nakitembe fruits had average β-CE levels of 16.9 and 20.9 µg/g dry weight (DW), respectively, which contrasts with findings by Mbabazi *et al*.,^15^ who reported mean values of 4.5 and 10.2 µg/g DW, respectively. However, the same study documented broad concentration ranges of 0.2 to 11.0 µg/g DW for M9 and 0.7 to 23.7 µg/g DW for Nakitembe, reflecting considerable environmental variability, as the samples were sourced from three distinct banana-growing regions in Uganda. All the wild-type controls in the present study were harvested exclusively from Buginyanya, a regulatory confined field trial site located in the cooler eastern highland region of Uganda (Bulambuli district). Despite the limited number of biological replicates in this study (*n* = 4), considerable variation in β-CE concentrations was observed among the wild-type controls, with values ranging from 14.3 to 19.4 µg/g dry weight (DW) in M9 and 14.2 to 27.6 µg/g DW in Nakitembe. In contrast, β-CE levels in the transgenic lines were consistently higher and generally exceeded the biofortification target of 20 µg/g DW. Specifically, transgenic M9–12083 ranged from 28.9 to 43.2 µg/g DW, M9–12141 from 17.5 to 24.6 µg/g DW, NKT-12431 from 40.2 to 68.5 µg/g DW, and NKT-12468 from 46.1 to 64.8 µg/g DW. These data highlight the stability and nutritional robustness of the transgenic events, with most lines maintaining β-CE levels above the target threshold across biological replicates. It is, however, well documented that environmental factors influence pVAC accumulation in banana fruit. For instance, Paul *et al*.^9^ reported that “Dwarf Cavendish” bananas took longer to mature during winter and in the process accumulated much higher levels of β-CE (8.1 µg/g DW) compared to their counterparts that quickly matured during summer (1.0 µg/g DW). Therefore, the elevated β-CE levels observed in the present study’s wild-type controls are consistent with an environmental effect on carotenoid accumulation. Future research should aim to quantitatively assess the influence of specific environmental parameters such as light intensity, precipitation, and temperature on pVAC biosynthesis and retention in banana fruit.

Traditional steaming of EAHBs resulted in β-CE losses at 8 h of up to 40.2% in transgenic line M9–12083 and 50.1% in line NKT-12468. In contrast, the corresponding wild-type cultivars exhibited comparatively lower reductions of 24.3% and 15.8%, respectively. These losses exceed the approximately 25% reduction previously reported by Mbabazi *et al*..^15^ Despite these substantial declines, β-CE concentrations at the end of the cooking period but also after averaging across all cooking time points, remained above the biofortification target threshold of 20 µg/g DW in all lines except M9–12141. The isolated failure of this single event to maintain β-CE levels above the target underscores the importance of selecting lead and backup events with significantly higher baseline β-CE concentrations to buffer against potential losses during food preparation. These findings also confirm that the effectiveness of a biofortification intervention will be genotype-dependent. While certain transgenic lines consistently deliver elevated pVA levels across variable cooking durations, others perform comparably to their wild-type, underscoring the need for line-specific evaluation in the selection of biofortified events for nutritional deployment. As such, it is recommended that all five independent transformation events per cultivar, currently undergoing multi-location regulatory field trials, be evaluated for β-CE retention following extended steaming. Events with marginally sub-optimal β-CE levels may still be considered for deregulation if cooking-induced losses are offset by enhanced bioavailability, as demonstrated in orange-fleshed sweet potato^29^ although this remains to be demonstrated in banana.

## Conclusion

In summary, this study provides the first comprehensive assessment of pVAC retention in biofortified EAHBs subjected to prolonged traditional cooking. Despite significant initial losses during the first hour of steaming, β-CE concentrations in most transgenic events remained above the established nutritional target of 20 µg/g dry weight, even after 8 h of cooking. These findings confirm the nutritional resilience of selected biofortified lines under culturally relevant preparation methods, addressing a major concern for biosafety regulators and end-users alike. With the potential to meet more than 50% of the recommended dietary allowance for vitamin A, these biofortified bananas represent a scientifically validated and culturally appropriate solution to combat VAD in banana-dependent populations. Accelerated regulatory approval and deployment are therefore warranted to unlock their full public health impact.
